# Acclimatization of *Cicer arietinum* L. plants to salinity: examining the relief role of autochthonous mycorrhiza and exogenous proline

**DOI:** 10.1186/s12870-025-07886-9

**Published:** 2025-12-26

**Authors:** Reda E. Abdelhameed, Rabab A. Metwally

**Affiliations:** Botany and Microbiology Department, Faculty of Science, Zagazig University, Zagazig, 44519 Egypt

**Keywords:** Chickpea, Glomalin, Glycine betaine, Membrane injury, Mycorrhiza, NaCl stress, Phosphatases

## Abstract

Chickpea (*Cicer arietinum* L.) plant, among the most widely grown worldwide crops, faces a formidable foe: salinity which impedes its growth. In this regard, an experiment was stated to evaluate the effect of arbuscular mycorrhizal fungi (AMF) inoculation and exogenous proline (100 ppm) application on the growth and metabolic constituents of chickpea plants under salt stress (200 mM) conditions. Six treatments were tested: control, NaCl, proline, NaCl + proline, AMF, and NaCl + AMF. All growth indicators, leaf water content (8.0%), chlorophyll content (53.0%) and membrane stability index (36.8%) showed a decrease in plants subjected to salinity stress while an increase in membrane lipid peroxidation (72.9%) was noted as versus the control plants; however, proline-applied and AMF-colonized plants showed the reverse responses. A discernible rise in total phenols (49.1 and 35.6%), flavonoids (66.5 and 38.5%) and phenylalanine ammonia-lyase (7.1 and 4.7%) was observed with AMF and proline application under salt stress. The most obvious result is the role of AMF in strengthening the contents of soil easily (70.0%) and total glomalin (78.3%) and root acid (26.5%) and alkaline (41.8%) phosphatases. These results demonstrate that AMF and proline improve physiological and biochemical performance of chickpea under salt stress, with AMF showing the strongest benefits. Importantly, the application of AMF or proline offers a practical and eco-friendly strategy to mitigate salinity stress in chickpea cultivation, supporting sustainable crop production in salt-affected soils.

## Introduction

Since their emergence, land plants have existed in a hostile environment and had to contend with a diverse of abiotic challenges, and salinity is one of these challenges that restrict the development, growth, and metabolism of plants [1, 2]. Approximately 10% of arable land and nearly one billion hectares worldwide are affected by salinity, with projections suggesting that up to half of global farmland may be at risk by 2050 [3, 4]. Excess salts, especially sodium chloride (NaCl), disrupt water uptake, ion homeostasis, photosynthesis, and metabolic balance, thereby threatening global food security [5].

Several strategies have been employed to mitigate salinity stress, including breeding for salt-tolerant varieties, improved irrigation practices, and the application of plant growth regulators, organic solutes and plant growth-promoting microorganisms [6, 7]. Among these, arbuscular mycorrhizal fungi (AMF) are considered efficient bio-ameliorators of salt stress and in addition to shielding host plants from the deleterious impacts of salinity, also improves their productivity [8, 9]. AMF establish symbiotic associations with the roots of most terrestrial plants [7], improving nutrient and water uptake, modulating rhizosphere conditions, and enhancing host tolerance to both abiotic and biotic stresses [10–12].

In recent years, there has been an increasing curiosity in using autochthonous AMF since these native isolates are naturally acclimated to the local soil and climate, which frequently improves their symbiotic performance and colonization efficiency when compared to commercial or model strains. As members of the native microbial community, autochthonous AMF differ from allochthonous inocula in that they are more ecologically compatible, more persistent in the rhizosphere, and more effective at promoting plant growth in response to site-specific stressors like salinity, drought, or nutrient deficiencies [13, 14]. Numerous studies have shown that because local AMF strains have a higher degree of functional compatibility and co-adaptation with the host plant, they frequently perform better than commercial consortia in enhancing nutrient uptake, biomass accumulation, and stress resilience of crops, including chickpea [15]. Furthermore, studies employing AMF for inoculation in chickpea soils affected by salinity have shown notable enhancements in growth, physiological health, and yield components, indicating that locally adapted fungi outperform commercial strains that are more widely distributed under abiotic stress [16]. Similarly, when native AMF strains, like those dominated by *Glomus* and *Acaulospora*, are included, site-specific inoculation experiments in chickpea show increased root colonization and improved phosphorus acquisition [17]. Consequently, autochthonous AMF are a valuable bioinoculant resource for sustainable agriculture because to their ecological significance and host-specific potential, especially for crops like chickpeas grown in marginally saline soils [13, 16].

In parallel, exogenous proline application has emerged as a cost-effective and environmentally friendly approach to salinity stress management [18, 19]. Several studies have demonstrated its capacity to improve seed germination, growth, and yield under saline conditions, though responses vary with species and growth stage. For instance, foliar proline treatment boosted plant growth and improved production characteristics in salt-stressed *Zea mays* L [20]. More recently, Koc et al. [19] claimed that proline builds up as a result of salinity and the overexpressing of it has been shown to improve the rice plants’ ability to withstand salinity. In celery and radish plants, proline treatment efficiently reduces salinity stress through growth promotion, maintenance of photosynthetic efficiency, preservation of cellular integrity, control of protein homeostasis, bolstering of antioxidant defense, and manipulation of osmotic balance and redox state [21, 22].

On a worldwide scale, escalating soil salinization causes serious abiotic stress on crops such as chickpeas (*Cicer arietinum* L.) [23]. This legume, which fellow the Fabaceae family, is the world’s third-most important pulse crop behind dry beans and peas and used for human consumption and animals fed. In terms of grain legumes, it provides a decent source of necessary amino acids, 61.5% carbohydrates, 4.5% fat, and 20–25% protein [24]. As well, chickpeas can fix atmospheric nitrogen naturally, which increases soil fertility. Despite their nutritional value, chickpeas are threatened by saline. Therefore, to enable chickpea plants to mitigate salt stress, more investigation is required to obtain comprehensive understanding of the AMF and proline-associated pathways which is the main aim of the present study. In addition, there is limited knowledge on the performance of autochthonous AMF isolates in chickpea under salinity, as well as their comparative effects relative to exogenous proline. Therefore, our primary objective of the present study is to address this gap by evaluating the effects of exogenous proline and autochthonous AMF separately on minimizing salt stress in chickpeas in order to establish baseline knowledge of their respective roles in enhancing their growth, physiological and biochemical traits. We hypothesize that the application of autochthonous AMF or exogenous proline will exert positive effects in improving salt stress tolerance in chickpea by enhancing water relations, total antioxidant defense, secondary metabolites and osmotic adjustment, thereby supporting sustainable cultivation under saline soils.

## Materials and methods

### Preparation of the AMF inoculum

In Minia Al-Qamh, El-Sharkia Governorate, Egypt, the AMF, *Gigaspora margarita*, *Funneliformis mosseae*, *Rhizophagus irregularis*, and *F. constrictum*, was previously isolated from the rhizosphere of different plant species (wheat, beans and other grasses) using wet sieving and decanting techniques in compliance with Gerdemann and Nicolson [25]. In summary, 500 g of rhizospheric soil was placed in a container, and then 10 L of water was added and thoroughly combined to form a suspension of soil and water. After settling for five minutes to exclude heavy and insoluble particles, the suspension was sieved using sieves with pore diameters of 500, 250, 150, and 38 μm in order to extract spores. The characteristics of asexual spore formations listed in an INVAM [26] handbook were used to identify the retrieved AMF spores. To increase the AMF population, the AMF spore mixture was cultivated for five months on the roots of Sudan grass (*Sorghum sudanenses*) in a sterile substrate of sandy clay soil as trap plants.

### Experimental design, treatments (NaCl, AMF and proline) and growth condition

Under greenhouse conditions, the experiment was carried out at a temperature around 23 °C, a photoperiod of 14 h, and a relative air humidity of 50–75%. Chickpea (*Cicer arietinum* L. Giza 531) seeds were provided from the Agriculture Research Centre, Ministry of Agriculture, Giza, Egypt. Sandy clay soil, pH: 7.4, was collected from Belbis, Sharkia Governorate. Pots of 24 cm diameter were containing 2.5 kg of sterilized soil in a greenhouse at Zagazig University’s Faculty of Science in Egypt. A full factorial 6 × 5 randomized pot trial with three components (proline, AMF, and NaCl) made up the experiment. There were 6 treatments including T1 (control), T2 (NaCl: 200 mM), T3 (proline: 100 ppm), T4 (NaCl: 200 mM + proline: 100 ppm), T5 (AMF) and T6 (NaCl: 200 mM + AMF) with 5 replicas. Concerning AMF inoculation, seeds were planted in pots which received, on the day of sowing, 25 g of AMF inoculum each pot (about 70 spores/g trap soil and infected root pieces, M = 75%). Sterilized soil, rather than any AMF inoculum, was given to the control treatments.

Chickpea plants (5 in each pot) were established for 3 weeks before salinization in order to facilitate proper plant growth and AMF association formation. Regarding NaCl treatments, the pots were applied with two concentrations of NaCl solution (0 and 200 mM), and for four weeks, the plants received twice-weekly irrigation. During NaCl addition, proline was added at 100 ppm in the irrigation water, three times in two weeks. After 4 weeks from NaCl treatments, sampling of chickpea plants was done for mycorrhizal, morphological and biochemical evaluations.

### Measurements of the response variables

#### Qualitative and quantitative assessments of mycorrhizal infection

Utilizing Phillips and Hayman [27], mycorrhizal root infection was measured. In a nutshell, fresh root samples were sliced into lengths of 0.5–1 cm. After being cleaned for ten minutes at 95 °C in 10% KOH, these root segments were stained. Under a light microscope at ×10 magnification, thirty stained root sections from each treatment were placed on slides, and the colonization was determined by calculating the percentage of these intersections that displayed particular fungal features (vesicles, intercellular hyphae, and arbuscules). Using the Mycocalc program (http://www.dijon-inra.fr/mycocalcprg), the Trouvelot et al. [28] approach was applied to determine the frequency (F%), intensity (M%), and rate (A%) of mycorrhizal colonization in the stained roots.

#### Lengths of shoots and roots, fresh and dry weights, and carotenoids and chlorophylls in leaves

After uprooting the entire plant, the lengths of the shoots and roots were measured with a measuring ruler. After measuring the fresh weight of the roots and shoots for each treatment, the same plants were dried for two days at 60 °C in a hot air oven to determine the dry weight. Using 85% acetone as an extraction solvent, leaf chlorophylls a, b, and carotenoids were measured using the Metzner et al. [29] method. Their concentrations were determined spectrophotometrically at specific wavelengths: chlorophyll a at 663 nm, chlorophyll b at 645 nm, and carotenoids at 480 nm, and expressed as mg g^− 1^ fwt.

### Measurement of the water status and membrane injury index

To evaluate the plant water status, water content (Wc) and water saturation deficit (Wsd) were calculated by Barr and Weatherley [30] method. The membrane stability index (MSI) is frequently used to quantify the damage that stress causes to membranes, and it was calculated following the technique outlined by Sairam [31]. About 1000 mg of leaves were made as tiny discs, which were then cleaned and left for 2.5 at 40 °C in a 30 mL of double-distilled water in a water bath. Using the electrical conductivity meter, the electrical conductivity (CI) of the discs was recorded. For 30 min, the sample discs was submerged in boiling water at 100 °C without being taken out, and the electrical conductivity (C2) was once more recorded. The MSI was computed as; MSI (%) = [1-(C1/C2)] × 100. Membrane injury (MI) was calculated using Dhanda et al. [32] as the ratio of stressed plants’ MSI to control plants’ MSI. MI (%) = [1− (MSI (stressed)/MSI (control)] × 100.

According to Heath and Packer [33], membrane lipid peroxidation (MLP) is the oxidative breakdown of lipid-fatty acids by reactive oxygen species (ROS) and is quantified by the amount of thiobarbituric acid reactive compounds. Fresh leaf tissue (0.5 g) was homogenized in 5 mL of 0.1% (w/v) trichloroacetic acid (TCA) and centrifuged at 8000 ×g for 10 min. An aliquot of the supernatant (1 mL) was mixed with 4 mL of 20% TCA containing 0.5% thiobarbituric acid. The mixture was heated at 95 °C for 30 min, rapidly cooled on ice, and centrifuged again at 8000 ×g. The absorbance of the supernatant was recorded at 532 nm and corrected for nonspecific absorbance at 600 nm. The molar coefficient (155 mM^− 1^ cm^− 1^) was used to calculate the MLP content, which was then represented as nmol g^− 1^ fwt.

#### Estimation of protein and glycine betaine

The content of soluble protein in chickpea roots was calculated using the Folin phenol reagent in accordance with Lowry et al. [34], and the absorbance was measured at 700 nm using bovine serum albumin as a reference. Using the colorimetric approach, the amount of glycine betaine in lyophilized fresh chickpea leaves was ascertained [35]. Mix the lyophilized tissue with 1.5 mL of 2 N H_2_SO_4_, heat it for 10 min at 60 °C in a water bath, and then mix it with 50 µL of cold KI-I_2_. Samples were held at 0–4 °C for 16 h, centrifuged for 15 min at 4 °C, and then placed on ice for an hour. Following the collection of the supernatant, 4.5 mL of 1, 2-dichloroethane was added. The absorbance of the combination at 365 nm was measured following two hours of incubation at room temperature.

#### Total antioxidant capability using the technique of phosphomolybdenum

The total antioxidant capacity of chickpea leaves was determined using the phosphomolybdenum method described by Prieto et al. [36]. Fresh leaf tissue (0.5 g) was homogenized in 5 mL methanol and centrifuged at 8000 ×g for 15 min. An aliquot of the supernatant (0.3 mL) was mixed with 3 mL of reagent solution containing 0.6 M sulphuric acid, 28 mM sodium phosphate dibasic, and 4 mM ammonium molybdate tetrahydrate. The reaction mixture was incubated in a boiling water bath at 95 °C for 90 min and then cooled to room temperature. The absorbance was recorded at 695 nm against a reagent blank. Antioxidant capacity was expressed as mg ascorbic acid equivalents g^− 1^ fwt, based on a calibration curve prepared with standard ascorbic acid solutions.

#### Determination of total phenols, flavonoids and phenylalanine ammonia-lyase

The quantitative estimation of total phenols was carried out following Folin–Ciocalteu assay [37, 38]. Fresh leaf samples (0.5 g) were extracted with 10 mL methanol and centrifuged at 8000 ×g, then 1 mL of the extract was mixed with 1 mL of Folin–Ciocalteu reagent and 1 mL of 20% sodium carbonate (Na_2_CO_3_). The reaction mixture was incubated, and absorbance was recorded at 650 nm using a spectrophotometer. Phenolic content was determined from a standard curve prepared with gallic acid.

Total flavonoids in fresh chickpea leaf tissue (0.5 g) were quantified using the aluminum chloride colorimetric assay as described by Chang et al. [39]. An aliquot of 100 µL of the ethanolic extract was diluted with 4 mL of distilled water. Subsequently, 0.3 mL of 5% sodium nitrite was added at time zero, followed by 0.3 mL of 10% aluminum chloride after 5 min. After an additional 6 min, 2 mL of 1 M sodium hydroxide was added to the mixture. Absorbance was measured at 415 nm against a reagent blank, and quercetin was used as a reference.

PAL activity was assayed cording according to McCallum and Walker [40]. Fresh leaf tissue (0.5 g) was homogenized in 5 mL of 50 mM borate buffer (pH 8.8) and centrifuged at 10,000 ×g for 15 min at 4 °C. The reaction mixture contained 0.5 mL of enzyme extract, 1.5 mL of borate buffer, and 1.0 mL of 10 mM L-phenylalanine. After incubation at 37 °C for 1 h, the reaction was stopped by placing tubes on ice, and the absorbance was recorded at 290 nm then PAL activity was expressed as units/min.

#### Phosphatases (acid and alkaline) activity

The Tabatabai and Bremner [41] method was used to measure the activity of both phosphatase enzymes in chickpea roots. Root tissues were crushed in 5 mL of ice-cold sodium acetate buffer (0.1 M, pH 5.0) for the acid phosphatase activity assay, and sodium phosphate buffer (100 mM, pH 8.0) for the alkaline phosphatase activity reaction. After centrifuging the resulting homogenate for 15 min, nitrophenyl phosphate disodium salt (NPP) was used as a substrate for the test. The activity was measured in µmol of nitrophenyl emitted per minute at 410 nm.

#### Glomalin (easily and totally extractable)

To determine the concentration of total and readily extractable glomalin-related soil proteins, soil samples were taken from the rhizosphere [42]. 2 g of soil samples were mixed with 8 mL of 20 mM sodium citrate (pH 7.0), autoclaved for 30 min at 121 °C, then centrifuged for 15 min at 6000 rpm in order to measure the readily extractable glomalin. For analysis, the supernatant was kept at 4 °C. As part of the whole glomalin extraction process, a second 2 g soil sample was autoclaved for 60 min with 8 mL of 50 mM sodium citrate (pH 8.0) and centrifuged for 15 min at 6000 rpm. Bovine serum albumin was used as a standard by Lowry et al. [34] to evaluate their contents.

### Statistical analysis

The means ± standard error of five replicas (*n* = 5) are the results displayed in the tables and graphs. Using the Statistical Package for Social Sciences (SPSS) software version 14, one-way analysis of variance (ANOVA) and Duncan’s technique were employed to determine whether there was a significant difference between the treatment and control groups. Pearson correlation coefficients, neighbor joining clustering and principal component analysis (biplot) were also carried out employing the Past software. The graphical presentation was done using Originpro 2017 for Graphing and Analysis.

## Results

### Mycorrhizal status and colonization percentages

**Fig. 1:**
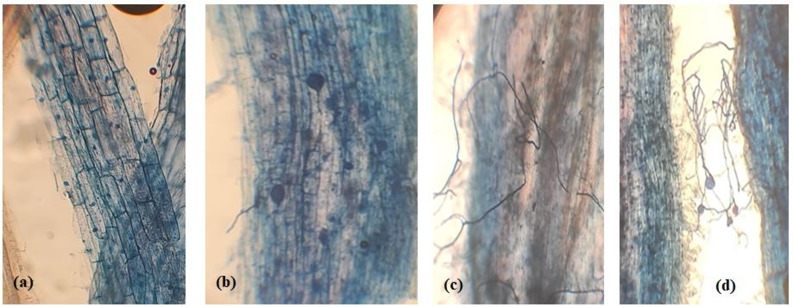
Photomicrographs of chickpea root cells; (**a**) control without colonization and (**b**-**d**) AMF-inoculated chickpea roots showing vesicles and hypha of AMF

AMF treatment and the salt × AMF interaction had a substantial impact on root colonization (Table 1). The colonization rate of AMF roots was noticeably higher at the 0 mM salt treatment which was reflected by the level of arbuscular formation in root segments (A%), frequency of root segments (F%) and mycorrhizal colonization intensity in root tissues (M%) where the percentages are 98.44a (F%), 56.23a (M%) and 21.29a (A%). Moreover, at 200 mM salt level, the root colonization was significantly decreased; 90.00b (F%), 32.00b (M%) and 10.19b (A%). Non-AMF inoculated control plants’ roots did not become colonized. Additionally, results in Fig. 1 demonstrated photomicrographs of the stained roots of chickpeas showing the structural colonization of AMF (vesicles and hypha).

**Table 1:** Effect of sodium chloride (NaCl) on mycorrhizal infection of chickpea root

NaCl	AMF	Proline	F (%)	M (%)	A (%)
0 mM	-	-	0^c^	0^c^	0^c^
	+ AMF	-	98.44^a^	56.23^a^	21.29^a^
	-	+100 ppm	0^c^	0^c^	0^c^
200 mM	-	-	0^c^	0^c^	0^c^
	+ AMF	-	90.00^b^	32.00^b^	10.19^b^
	-	+100 ppm	0^c^	0^c^	0^c^

Values are the mean of 5 replicas. Different ^abc^Superscripts in each column indicate differences between treatments based on Duncan test (*p* ≤0.05). F%: frequency of root segments, M%: mycorrhizal colonization intensity in root tissues and A%: arbuscular formation in root segments

### Lengths of the shoots and roots, fresh and dried weights

**Fig. 2:**
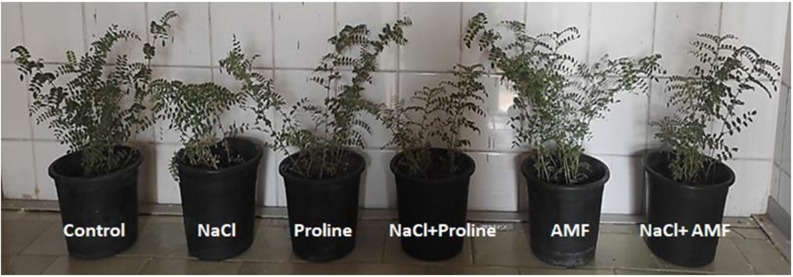
Chickpea plants under the control and different treatments [sodium chloride (200 mM NaCl), proline, arbuscular mycorrhizal fungi (AMF) and their combinations]

**Fig. 3:**
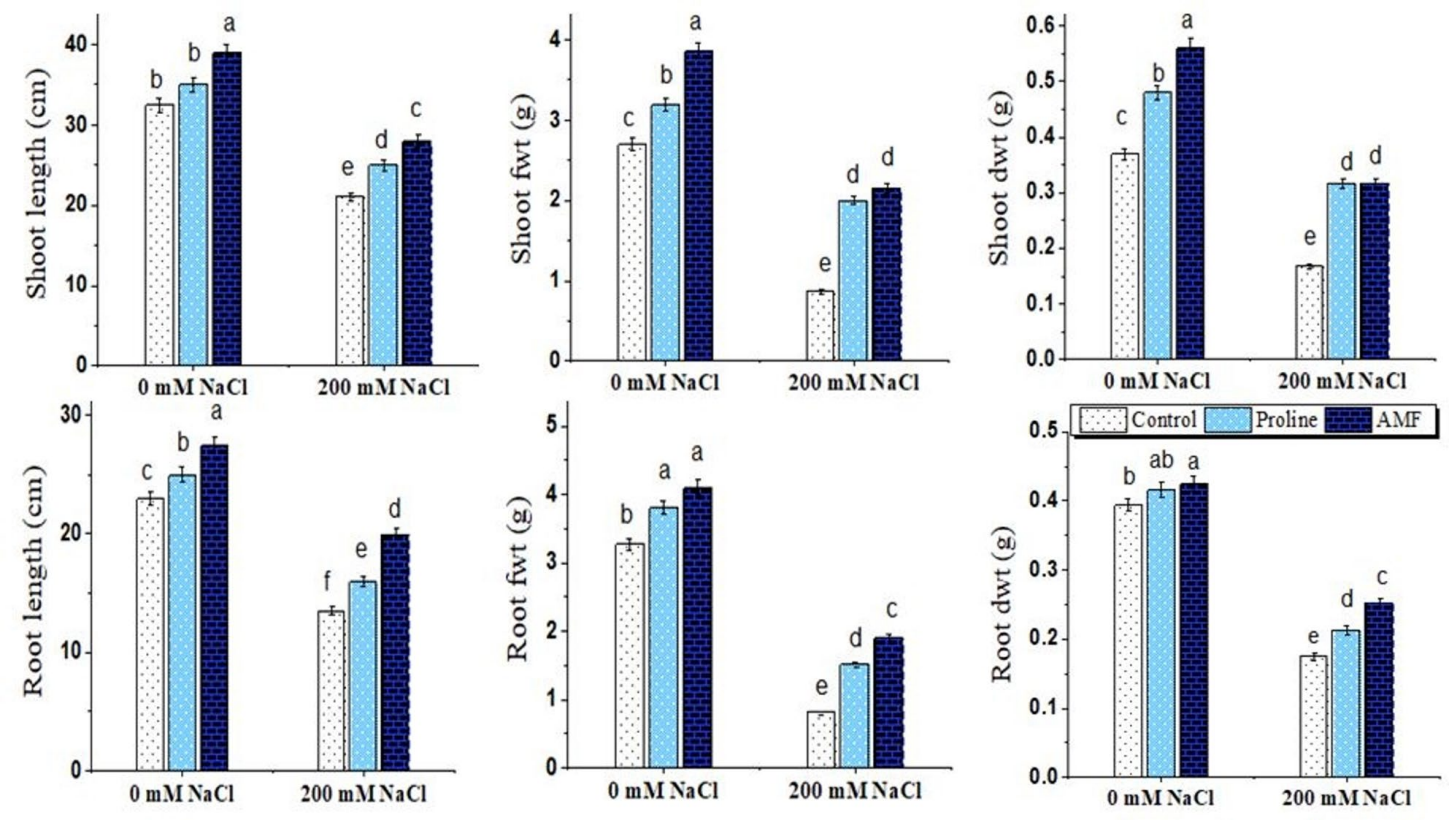
Effect of proline, arbuscular mycorrhizal fungi (AMF) and sodium chloride (0 and 200 mM NaCl) on shoot and root growth parameters of chickpea plants. Values are the mean ± standard error (5 replicas). Different ^abc^Superscripts above columns indicate differences between treatments based on Duncan test (*p* ≤ 0.05)

In the present study, NaCl, AMF inoculation, and proline application affected the length, weights of the roots and shoots, both fresh and dry of chickpea plants (Fig. 2 and Fig. 3). It was detected that salinity stress significantly suppressed all the growth parameters of chickpea plants relative to the control with a reduction of 35.4% for shoot length, 41.3% for root length, 68.1% for shoot fresh weight and 75.5% for root fresh weight. However, with AMF colonization or proline application, length, fresh and dry weights significantly enhanced in the salt-stressed chickpea compared to their corresponding salt-stressed ones.

### Chlorophylls and carotenoids

**Fig. 4:**
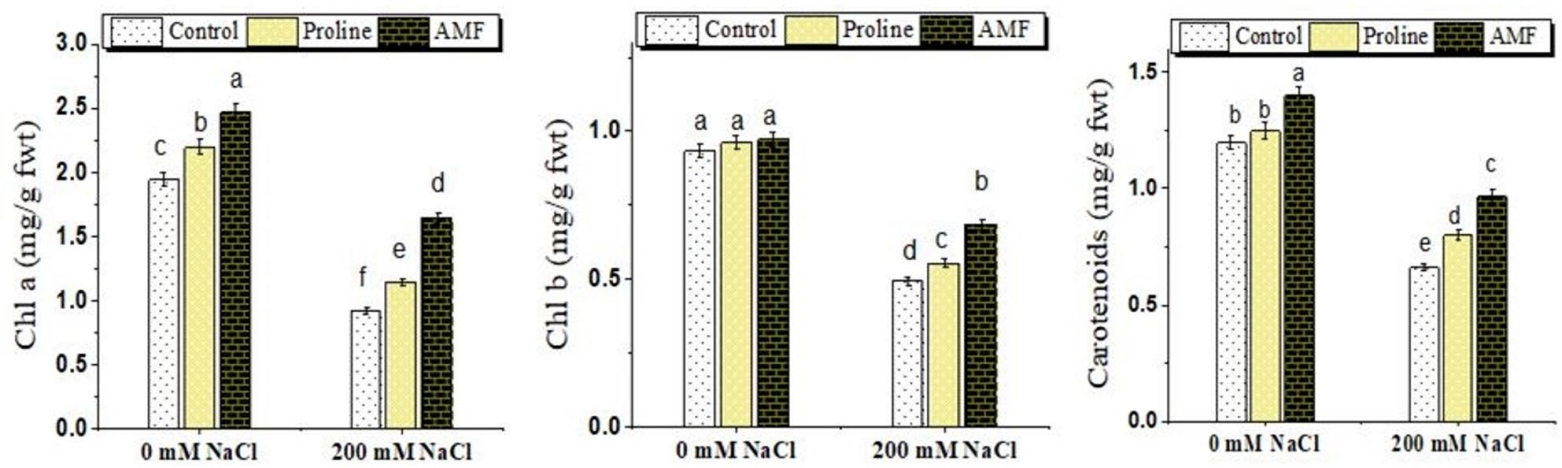
Effect of proline, arbuscular aycorrhizal fungi (AMF) and sodium chloride (0 and 200 mM NaCl) on chlorophyll a (Chl a), chlorophyll b (Chl b) and carotenoids content of chickpea plants. Values are the mean ± standard error (5 replicas). Different ^abc^Superscripts above columns indicate differences between treatments based on Duncan test (*p* ≤ 0.05)

According to one-way ANOVA results, chlorophyll and carotenoids content was significantly impacted by NaCl, AMF treatment and proline application (Fig. 4). NaCl (200 mM) resulted in a reduction of chlorophylls a, b and carotenoids with a percent of decrease; 52.80, 47.10 and 44.78% respectively. Interestingly, mycorrhizal symbiosis or proline application increased these pigment fractions in chickpea leaves (Fig. 4) under control and salt-stressed conditions. Under control conditions, AMF and proline increased chlorophyll a by 26.1 and 12.7%, and chlorophyll b by 4.0 and 3.2%. Under salt conditions, AMF and proline significantly raised chlorophyll a by 78.8 and 24.6%, chlorophyll b by 38.7 and 12.1%, and carotenoids by 20.5 and 46.2% compared with salt-stressed plants.

### Water status and membrane damage traits

The consequence of NaCl, AMF and proline on water status (Wc and Wsd) and membrane traits (MSI and MI) was tabulated (Table 2). When chickpea plants were subjected to 200 mM NaCl, their Wc and MSI significantly dropped with a reduction percent of 8.0 and 36.8% respectively. This impact was lessened by application of AMF and proline in the chickpea plants where, under stress conditions, AMF colonization increased Wc by 8.27% and MSI by 70.60% compared to untreated plants.

**Table 2:** Effect of proline, arbuscular mycorrhizal fungi (AMF) and sodium chloride (0 and 200 mM NaCl) on membrane stability index (MSI), membrane injury (MI), water content (Wc) and water saturation deficit (Wsd) of chickpea plants

NaCl	AMF	Proline	MSI (%)	MI (%)	Wc (%)	Wsd (%)
0 mM	-	-	71.67 ± 1.89^a^	-	85.58 ± 2.237^a^	18.36 ± 0.485^c^
	+ AMF	-	77.27 ± 2.04^a^	-	85.21 ± 2.254^a^	9.50 ± 0.251^e^
	-	+ 100 ppm	74.95 ± 1.98^a^	-	87.59 ± 2.238^a^	14.13 ± 0.374^d^
200 mM	-	-	45.28 ± 1.19^d^	36.83 ± 0.974^a^	78.71 ± 2.067^b^	34.76 ± 0.919^a^
	+ AMF	-	65.80 ± 1.74^b^	14.85 ± 0.393^c^	83.04 ± 2.302^a^	22.92 ± 0.61^b^
	-	+ 100 ppm	58.96 ± 1.56^c^	21.33 ± 0.564^b^	84.71 ± 2.267^a^	23.72 ± 0.627^b^

Values are the mean ± standard error (5 replicas). Different ^abc^Superscripts in each column indicate differences between treatments based on Duncan test (*p* ≤ 0.05)

**Fig. 5:**
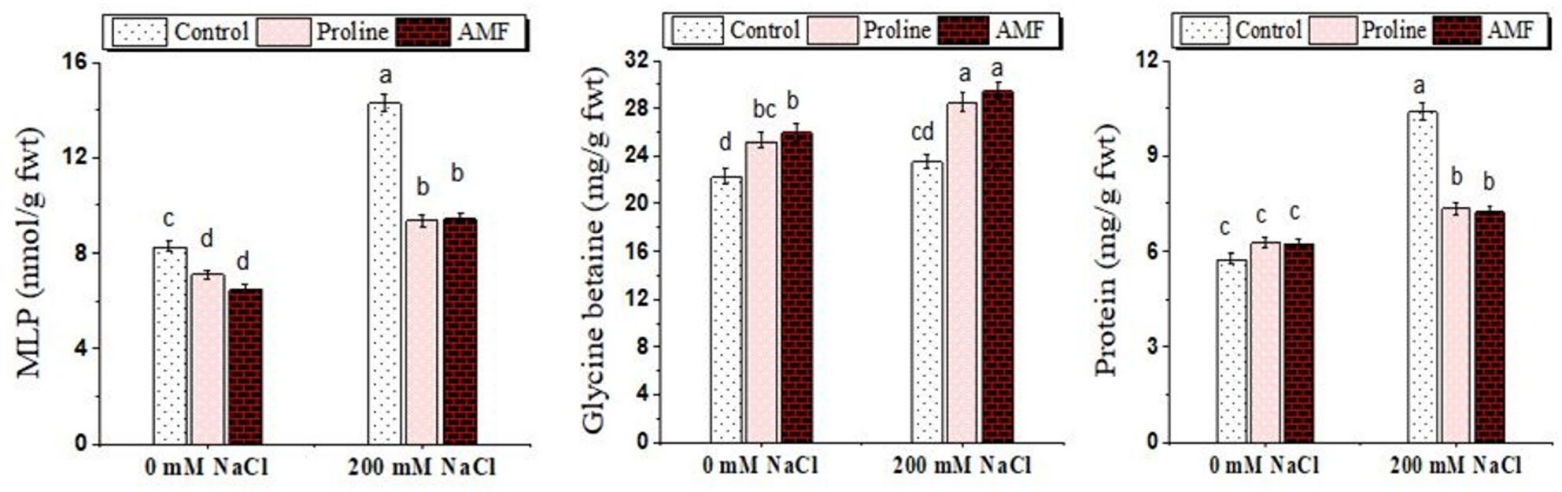
Effect of proline, arbuscular mycorrhizal fungi (AMF) and sodium chloride (0 and 200 mM NaCl) on membrane lipid peroxidation (MLP), glycine betaine and protein contents of chickpea plants. Different ^abc^Superscripts above columns indicate differences between treatments based on Duncan test (*p* ≤ 0.05)

Results in Fig. 5 displayed that MLP (nmol g^− 1^ fwt) was significantly increased in plants grown under 200 mM NaCl concentration (14.32a) relative to the control ones (8.28c). However, a lower MLP concentration was detected in AMF-colonized plants (6.50 cd) and proline applied plants (7.13d). It is obvious that the MI decreased significantly from 36.83% in non-inoculated stressed plants to 14.85% in AMF inoculated plants and 21.33% in proline applied plants **(**Fig. 5**)**.

### Osmolytes (protein and glycine betaine)

The study’s findings show how the protein and glycine betaine content of chickpea plants is affected by the administration of AMF and proline, either with or without NaCl. Our results revealed that NaCl significantly induced protein and glycine betaine accumulation (10.42 and 23.55 mg g^− 1^ fwt) compared to control (5.77 and 22.31) (Fig. 5). A noteworthy outcome is that a further increase in their concentration was observed with AMF symbiosis (6.24 and 26.00) and proline applications (6.29 and 25.32) under non-NaCl stress. Conversely, under NaCl stress, AMF and proline application showed a decrease in the soluble protein content of chickpea plants compared to NaCl-stressed plants only. While a significant increment in glycine betaine level under NaCl stress was evidenced with AMF colonization and proline application.

### Total antioxidant capacity

**Fig. 6:**
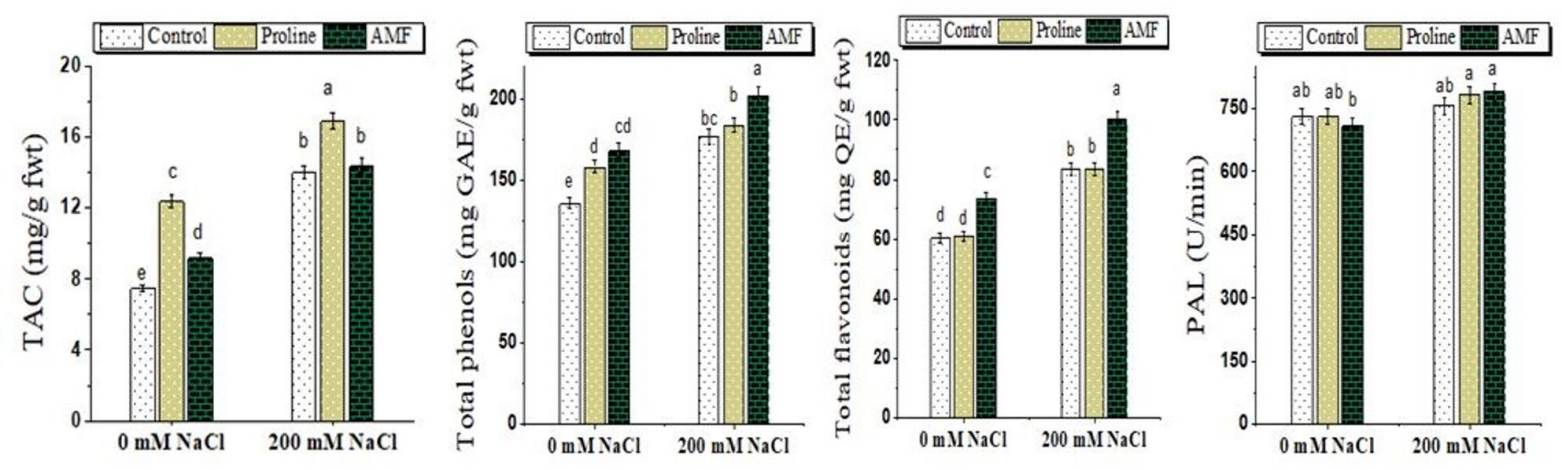
Effect of proline, arbuscular mycorrhizal fungi (AMF) and sodium chloride (0 and 200 mM NaCl) on total antioxidant capacity (TAC), total phenols, total flavonoids and phenylalanine ammonia-lyase (PAL) of chickpea plants. GAE: gallic acid equivalent, QE: quercetin equivalent. Different ^abc^Superscripts above columns indicate differences between treatments based on Duncan test (*p* ≤ 0.05)

As compared to the control and according to the findings in Fig. 6, the amounts of total antioxidant capacity in chickpea plant leaves under 200 mM NaCl conditions were significantly greater. Notably, under stress, a further and significant increase in total antioxidant capacity with AMF application (14.39) and proline (16.88) was detected compared to the stressed plants only (13.98 mg g^− 1^ fwt).

### Total flavonoids, total phenols, and phenylalanine ammonia-lyase

In chickpea leaves subjected to 200 mM NaCl stress, total phenols, flavonoids, and phenylalanine ammonia-lyase reached 177.22 mg GAE g^− 1^ fwt, 83.31 mg QE g^− 1^ fwt and 757.30 U/min respectively, compared with 135.77, 60.14 and 731.68 in untreated control plants. Remarkably, according to ANOVA findings, salt-stressed chickpea plants inoculated with AMF or treated with proline revealed a noteworthy and further upsurge in their contents over stressed plants only (Fig. 6).

#### Phosphatases

As significant hydrolytic enzymes that are extensively found in plants, phosphatases were determined in this study (Table 3**)**. Acid phosphatase activity was significantly greater than that of alkaline phosphatase, and their activities were extremely affected to a greater extent by AMF colonization and to a lower extent by proline application. Acid and alkaline phosphatase activities increased by 8.7% and 17.1%, in chickpea plants subjected to 200 mM NaCl. In AMF-colonized plants, a further enhancement was recorded, showing increases of 22.9% and 34.3% under non-stressed conditions and 15.5% and 21.2% under NaCl stress, compared with their respective controls.

**Table 3:** Effect of proline, arbuscular mycorrhizal fungi (AMF) and sodium chloride (0 and 200 mM NaCl) on activity of phosphatases (nmol PNP/min) and glomalin content (µg/ g soil) of chickpea plants

NaCl	AMF	Proline	Acid phosphatase	Alkaline phosphatase	Total phosphatase	Easily extractable glomalin	Total extractable glomalin
0 mM	-	-	195.35 ± 5.168^c^	75.27 ± 1.99^c^	270.63 ± 7.16^d^	54.64 ± 1.42^e^	73.86 ± 1.99^d^
	+ AMF	-	240.11 ± 6.35^a^	101.11 ± 2.67^a^	341.21 ± 9.03^ab^	71.92 ± 1.87^b^	101.24 ± 2.67^b^
	-	+ 100 ppm	212.85 ± 5.63 ^bc^	84.52 ± 2.236^b^	297.38 ± 7.86^c^	64.74 ± 1.68 ^cd^	81.44 ± 2.24^c d^
200 mM	-	-	213.94 ± 5.66 ^bc^	88.11 ± 2.33^b^	302.05 ± 7.99^c^	61.63 ± 1.61^d^	79.30 ± 2.33^d^
	+ AMF	-	247.19 ± 6.54^a^	106.77 ± 2.82^a^	353.96 ± 9.36^a^	92.89 ± 2.42^a^	131.73 ± 2.83^a^
	-	+ 100 ppm	229.61 ± 6.07^ab^	90.11 ± 2.38^b^	319.72 ± 8.45 ^bc^	69.20 ± 1.81 ^bc^	88.62 ± 2.38^c^

Values are the mean ± standard error (5 replicas). Different ^abc^Superscripts in each column indicate differences between treatments based on Duncan test (*p* ≤ 0.05)

### Glomalin rich soil protein

Soil glomalin concentrations were impacted by proline treatment, AMF inoculation, and NaCl, as indicated in Table 3. AMF had a considerable impact; in chickpea treated with 200 mM NaCl, levels of both completely and extractable glomalin raised by 66.1 and 50.7%, respectively, while AMF inoculation increased levels by 37.1 and 31.6%. However, the effect of proline treatment was not statistically significant.

#### Pearson correlation matrix, neighbor joining clustering and principal component analysis

**Fig. 7:**
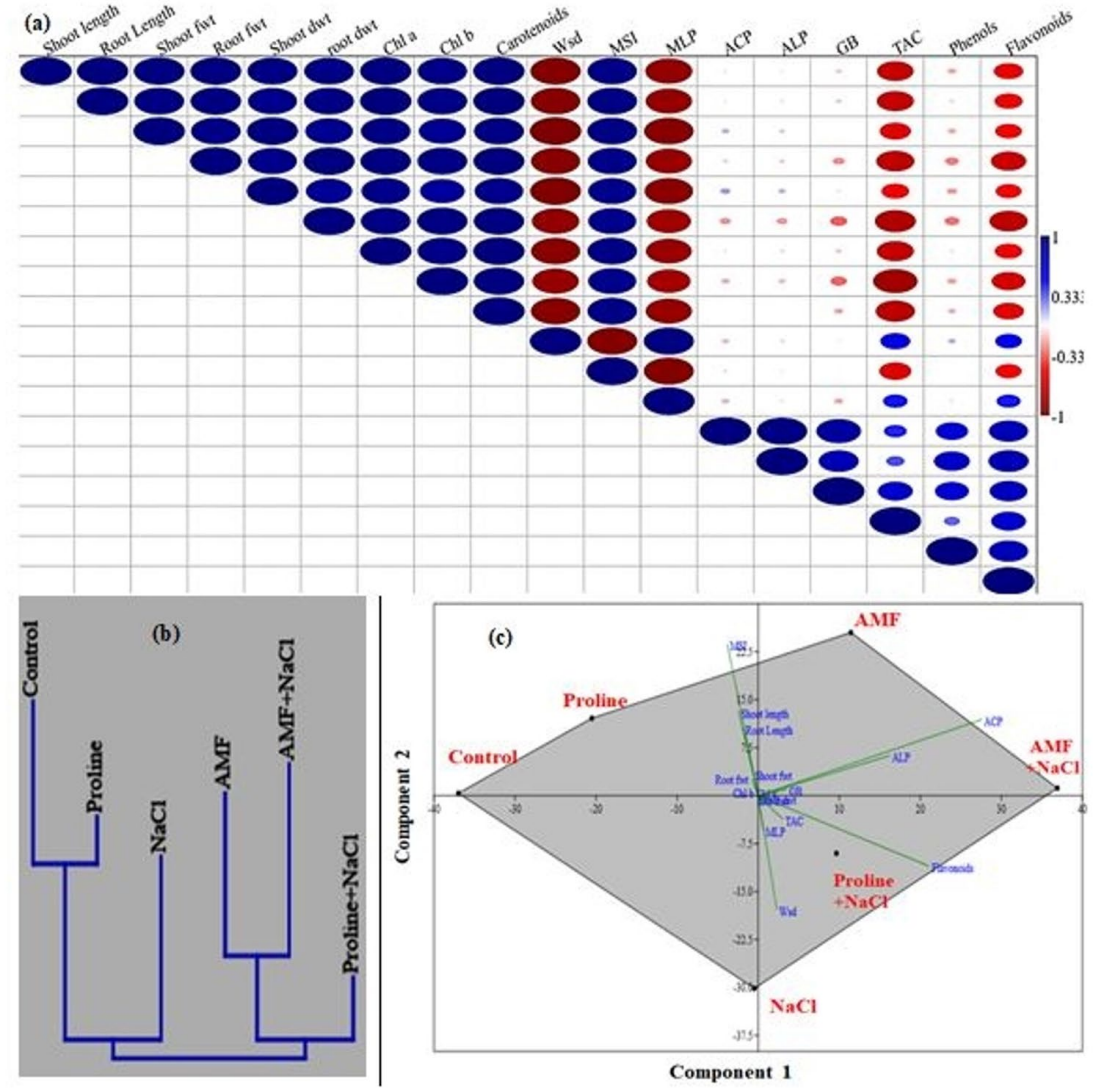
**a** Pearson’s correlation matrix of 18 traits in chickpea plants. Note: The intensity of color ranges from blue (positive) to red (negative) and the size of the circles show strength of significant correlation (*p* ≤ 0.05), **b** Neighbor joining clustering between six different treatments and (**c**) Principal component analysis (biplot) between the different treatments (red) and the studied parameters (blue) of chickpea plants. Note: TAC: total antioxidant capacity, ALP: alkaline phosphatase, ACP: acid phosphatase, GB: glycine betaine

The correlations between some of the assessed attributes were examined using a Pearson correlation matrix **(**Fig. 7a**)**. Significant correlations, both positive and negative, were found for the majority of the attributes examined. The most significant finding is that a noteworthy positive connection was observed between the measured levels of carotenoids, chlorophyll a, and b, as well as MSI, and all growth indices, including shoot and root lengths and fresh and dry weights of both shoots and roots. Furthermore, there were negative associations between MLP and Wsd and all shoot and root growth indices, carotenoids, chlorophyll a, and b, and MSI. This implies that boosting membrane stability, carotenoids, and chlorophyll are crucial elements in enhancing development characteristics.

To ascertain the relations between the different six treatments, neighbor joining clustering (Fig. 7b) was plotted. Next, a principal component analysis was then conducted in order to reduce the greatest amount of original data into principal components (PC). Figure 7c showed that 97.22% of the variance in the dataset was explained by the cumulative proportions of the first and second components (PC1 and PC2). In the collected data, PC1 described 63.56% of the variance, while PC2 provided 33.66% of the total variation. All of the treatments were effectively dispersed *via* the first two main components. The PCA biplot’s different parameters were most obviously separated into multiple clusters. Measures like MLP and Wsd were strongly separated and were close to the NaCl stress treatment. Other measures like MSI and some growth features were comparable to those of the control, proline and AMF treatments. Most clearly that, when NaCl stress is present, proline and AMF treatment was closely matched by total antioxidant capacity, glycine betaine and flavonoids which confirm the role of proline and AMF in lessening salt stress in chickpea plants *via* increasing antioxidant.

## Discussion

Chickpea is a sensitive genus to salt stress, like the other members of grain legumes [43]. The deleterious influences of saline soil on plant growth and development are linked to specific ion effect, low soil solution osmotic potential (water stress), nutritional imbalance that results in morphological, physiological, and biochemical alterations, such as decreased growth, ion imbalance, chlorophyll degradation, decreased photosynthesis and water status, and increased hydrogen peroxide [44]. In the pursuit of more sustainable agriculture, it is crucial to advance our understanding of the role of plant biotechnology. Therefore, to ensure food security, it is necessary to investigate various methods for improving the development of chickpea plants and making them more resistant to salt stress. In this study, we evaluated the potential of AMF species inoculation and proline treatment for mitigating salt stress in chickpea.

### AMF symbiotic development in chickpea roots

A sufficient symbiotic relationship between mycorrhiza and chickpea plant roots was confirmed by a greater colonization rate in non-NaCl, AMF-treated chickpea plants. Additionally, at 200 mM NaCl, there was a decrease in root colonization, indicating that salt interferes with the growth of AMF as well as the host plant. A study by Baltazar-Bernal et al. [45] supported these findings by showing that AMF-inoculated Taro plantlets under non-saline conditions had the highest colonization percentages, followed by plantlets with AMF + NaCl, whereas plantlets without AMF did not exhibit any colonization. Furthermore, studies on liquorice found that salinity significantly decreased *F. mosseae’s* capacity for roots colonization [46]. According to Sheng et al. [47] and Chaichi et al. [48], salinity suppresses the AMF symbiotic effect by lowering the colonization rate and having a direct harmful effect on AMF through Na^+^. High salt stress can inhibit hyphae growth; prevent spore germination, lower spore density, and lower spore viability, according to Ahanger et al. [49]. Additionally, it can reduce plant vesicles, arbuscules, and mycelium growth [7].

### Shoot and root lengths, fresh and dry weights

Chickpea plants’ fresh and dry weights, as well as their shoot and root lengths, are considerably decreased by salt (200 mM NaCl) stress. Under this stress, plants are exposed to harmful Na^+^ ions that damage cell organelles and interfere with metabolism, which hinders plant growth [50] that cause a reduction in plant height due to its adverse consequences on the rate of photosynthesis, growth hormones and enzyme activities [51, 52]. Rahman et al. [53] observed a harmful effect on seedling height and growth of chickpeas due to less mobilization of reserve food material, suspending the cell enlargement, cell division and injury during salinity. Also, the leaf senescence in chickpeas treated with NaCl may indicate that older leaves exhibit the effects of salt stress toxicity earliest, most likely as a result of transpiring longer and accumulating more Na^+^ in their tissues, which ultimately results in leaf mortality [45]. AMF-inoculated or proline-treated plants mitigated the NaCl effects on growth parameters by encouraging elongation and accumulation of biomass as Inayat et al. [22] and Yaqoob et al. [54] indicated. Because it functions as an osmolyte, controlling water balance and turgor pressure, proline helps radish plants under salt stress to maintain shoot and root length as well as plant fresh and dry weight [22]. This promotes root and shoot growth even in stressful situations [55]. Likewise, Baltazar-Bernal et al. [45] found an upsurge in the growth of Taro (*Colocasia esculenta* L.) plantlets with AMF + NaCl which is most likely caused by not only to storage of ion in the microcosms but also to less Na^+^ translocation. As AMF resides in the plant’s tissues and may result in a comparatively higher growth rate than the control treatment because of better water and minerals absorption through the external mycelium and a reduction in the accumulation of Na^+^ in the plant’s aerial sections.

### Chlorophylls and carotenoids

Prolonged exposure to salt stress causes a notable build-up of the key harmful ions, which causes chlorosis and leaf bleaching that lowers the photosynthetic ability of the plants. NaCl (200 mM) caused a reduction of chlorophyll and carotenoid in chickpea, which might be explained by the harmful consequences of Na^+^ and Cl^−^ ions. In addition, the production of proteolytic enzymes, like chlorophyllase, which disrupts the photosynthetic machinery and degrades chlorophyll, causing instability in the pigment-protein complex in plants [52, 56, 57]. Fascinatingly, these pigments in AMF-inoculated chickpea plants were superior to those of non-inoculated ones in both non-NaCl and NaCl-stressed soil as AMF inoculation diminishes the salt detrimental effects on photosynthetic pigments. This is in line with Metwally and Abdelhameed [58] findings, which may be endorsed to that mycorrhiza increases stomatal conductance, which enhances CO_2_ assimilation, and the availability of N and cofactors required in chlorophyll synthesis, such as Mn, Mg, and Fe, which are crucial parts of the electron carriers in the photosynthetic apparatus [59]. According to a study on *Echinacea angustifolia*, under salt stress, *R. irregularis* inoculation enhanced the chlorophyll content of leaves two to three times higher than treatments without AMF, as AMF improves the chlorophyll synthetase enzyme activity and eliminates toxic Na^+^ and hinders its transfer to aerial Sect [60]. Concerning the proline effect, it preserves chlorophyll and carotenoid levels in chickpea plants under normal and NaCl stress, as it helps in maintaining carotenoid and chlorophyll amounts, which are necessary for photosynthesis by maintaining pigment production pathways and stabilizing thylakoid membranes. Chickpea plants treated with proline or AMF exhibited higher carotenoids content and a decrease under NaCl. The rise in its content as a mechanism for resistance may reduce the photodegradation and photoinhibition of these photosynthetic pigments [59]. This guarantees ideal energy transfer and light absorption, both of which are essential for photosynthesis [55].

### Water status and membrane injury traits

Membrane injury traits give an idea about the sensitivity of cell membranes under a given set of stress conditions, as it is related to leaf tissue membrane stability. The present investigation found that cellular dehydration and membrane breakdown brought on by salt stress are indicated by decreased Wc and MSI and increased Wsd and membrane damage (MI and MLP). Borzouei et al. [61] stated a rise in cell membrane injury while a decrease in MSI under salt stress, which links back to a high extent of MLP caused by ROS [62]. Application of proline increases membrane integrity and water retention (Wc) and decreases MLP, as it improves the plant’s capacity to hold onto water and lessens the electrolyte leakage under salt stress [63]. Additionally, it keeps cellular proteins from being oxidatively damaged and aids in maintaining protein levels within the cells. As an osmoprotectant, proline aids in scavenging lipid peroxidation products, protects cellular structure and function, and increases the antioxidant enzyme activities, which scavenges ROS and preserve cellular redox equilibrium [64]. Concerning AMF effects in increasing Wc and reducing MLP in this study, Ruiz-Lozano [65] attributed these effects to the role of AMF in improving physiological functions such as enhancing hydraulic conductivity, favorably regulating the osmotic balance to increase the water absorption capacity of plants. Furthermore, AMF plants have a larger stomatal conductance, which raises the need for transpiration [47, 66]. Also, lowering Wsd in AMF-inoculated plants improved the water status [67]. The current results are consistent with Jahromi et al. [66], who discovered that regardless of salt level, lettuce plants infected with *G. intraradices* retained higher Wc than non-inoculated plants.

### Glycine betaine and protein

The protein and glycine betaine contents in chickpea plants have increased under NaCl stress, which are in line with the results of Baltazar-Bernal et al. [45]. AMF and proline application further induced glycine betaine accumulation. Corroborating these results, Abbaspour et al. [68] stated that the glycine betaine content improved significantly in *Pistachio vera* inoculated with *R. irregularis* and exposed to 250 mM NaCl, which contributed to improved salt tolerance by preserving the osmotic balance. Proline and glycine betaine biosynthesis and accumulation as protective and non-toxic osmolytes are stress-inducible [69], and their accumulation is proportional to the level of salt tolerance. Glycine betaine carries out a number of tasks, such as resilience to abiotic stress, and can also shield the photosystem II complex and Rubisco’s enzymatic activity during photosynthesis [70].

Regarding protein content, its increase under salt stress may be attributed to the induction of stress-responsive proteins such as heat shock proteins which play essential roles in osmoprotection, protein stabilization, and stress signaling [71]. Additionally, apparent increases in protein concentration can partly result from cellular dehydration and water loss, which concentrate existing proteins in the cytosol. However, under prolonged or severe salt stress, protein degradation and reduced protein synthesis have been widely reported, reflecting progressive damage and energy reallocation towards stress defense [72].

In the present study, AMF and proline treatments appeared to trigger an increase in soluble protein levels in salt stressed chickpea plants compared to control (non-salt stressed) that have been reported in numerous recent studies, indicating that these treatments support osmotic adjustment and protein homeostasis. For instance, when exposed to salt treatments, AMF-plants usually exhibit higher levels of soluble protein in their leaves or roots than non-AMF controls [73, 74]. Also, exogenous proline has been demonstrated to raise total soluble protein and maintain cellular integrity in saline environments due to osmoprotection and the stabilization of enzymes and membranes [75, 76]. However, under NaCl stress, AMF and proline treatments appeared to trigger a reduction in soluble protein levels compared to non AMF-inoculated and proline-treated plants. This suggests that the protective effect of AMF and proline in chickpea under salinity is mediated also through other osmotic adjustments and antioxidant defense.

### Phosphatases

Since 20–30% of the total P in soils is made up of organic phosphates, their dissolution by phosphatases should add to the pool of soluble P accessible for root uptake and enhance P nutrition [77]. The current findings demonstrated that acid and alkaline phosphatases were significantly increased in chickpea plants under 200 mM salinity; these results coincide with Xie et al. [78] and Abdelhameed et al. [79]. This could be because the pre-existing phosphatases are very resistant to stress-induced destruction or stress-stimulated synthesis of new phosphatases [58, 80]. Furthermore, both enzymes were more active after AMF inoculation, which may be closely linked to AMF colonization, as AMF reduce deficiencies in P and other minerals and increase phosphatase activity helps hydrolyze P into a form that plants can use [81]. Tisserant et al. [82] showed that alkaline phosphatase activity within AMF structures serves as a marker for fungal metabolic activity, whereas histochemical studies have demonstrated that active AMF infections exhibit notable ALPase activity, reflecting the fungi’s role in P metabolism. Also, proline can modulate phosphatase activity by maintaining ionic balance and protecting enzyme function against ROS accumulation.

### Total antioxidant capacity, total phenols, flavonoids and phenylalanine ammonia-lyase

Under salinity stress, plants synthesize enzymatic and non-enzymatic (e.g., flavonoids, total phenols) antioxidants that reflect the plant’s capability to neutralize oxidative stress as tolerance mechanisms [83]. The availability of phenolic hydrogens as scavengers of hydrogen-donating radicals and, as a result, a boost in antioxidant capacity for scavenging activity are characteristics of phenolic compounds [84]. Phenols and flavonoids are also essential for protecting against stress by reducing the chemical alterations and oxidative burst caused by salt stress in chickpea plant leaves. Likewise, to phenol content, chickpea plants showed an increase in antioxidant capacity and the highest content was obtained in AMF and proline-treated plants under NaCl stress. A study by Metwally and Soliman [52] and Abdelhameed et al. [85] showed a noticeable increase in the total phenols and flavonoids in tomato and fenugreek plants exposed to NaCl stress. In celery, exogenous proline application under salt stress environments resulted in enhanced growth, enhanced levels of flavonoids and total phenolics, as well as enhanced antioxidant capacity, which enhances salt tolerance [21]. The increased total phenols in AMF-colonized plants have been stated [86]. Djighaly et al. [87] found that the total antioxidant capacity rose greatly in *Casuarina equisetifolia* and *C. obesa* when inoculated with *R. fasciculatus* and exposed to 200 mM NaCl. Also, Baltazar-Bernal et al. [45] observed that the Taro plantlets grew more when treated with AMF + NaCl because of their increased tolerance to salt, which was connected to their increased soluble phenol content and antioxidant activity.

Also, the enhanced phenylalanine ammonia-lyase activity indicates its potential role in lessening salt stress in chickpea plants. It recruits the phenylpropanoid synthesis pathway, which forms the majority of secondary metabolite groups, regulates polyphenols, and is the main source of phenolic and flavonoid synthesis [88]. In this study, both mycorrhizal colonization and exogenous proline application can positively influence phenylalanine ammonia-lyase activity in chickpea plants. This enhancement contributes to increased phenolic compounds synthesis, improved antioxidant defenses, and greater tolerance to environmental stresses. AMF colonization has been observed to elevate phenylalanine ammonia-lyase activity, for instance, inoculation of *Piper nigrum* with *Claroideoglomus etunicatum* and *R. clarus* resulted in its increased activity, which was associated with higher phenolic content and improved plant defense mechanisms [89]. In *Moringa oleifera* L. trees subjected to salinity stress, proline foliar application led to increased phenylalanine ammonia-lyase activity, which was associated with enhanced growth, photosynthetic pigments, and overall stress resilience [90].

### Glomalin content

In our study, a notable effect in glomalin content was due to AMF inoculation, while a non-significant one was shown due to proline application. Glomalin is a highly stable glycoprotein released by AMF hyphae and spores, which remains in the soil, suggesting that it is a putative gene product of AMF origin. There are two types of it: readily extractable glomalin and total glomalin [91]. It is noteworthy that AMF-inoculated plants have an additional advantage over their non-AMF counterparts because salinity causes glomalin to accumulate in participating AMF, as demonstrated by our findings that AMF inoculation increased levels of both total and easily extractable protein [92]. Glomalin, produced in the hyphal wall and released into the environment, can form an insoluble, protective, glue-like layer covering the fungal hyphae and soil aggregate surfaces [91, 93]. It is necessary for the accumulation of soil organic carbon, for improving soil water and temperature, for stabilizing soil aggregates, and for regulating plant growth and community development. It was described as a heat shock protein 60 (HSP60) homolog [94] and is thought to help reduce cytosolic damage from Na^+^-mediated protein misfolding, preserve soil structure stability, and increase water-holding capacity, all of which contribute to an increased plant water content [95]. Salinity and glomalin production are strongly correlated, which furthers our knowledge of how AMF protect plants from salt stress [92].

This study’s findings are consistent with those of Garg and Baher [96], who reported that *G. mosseae* inoculation enhanced salt stress tolerance in chickpea where there was an increase in proline biosynthesis (increasing -pyrroline-5-carboxylate synthetase and glutamate dehydrogenase activities while decreasing proline dehydrogenase activity). However, the present work makes two novel contributions: (i) it uses autochthonous AMF isolates as comparative with proline application, demonstrating their positive effects, and (ii) it evaluates multiple additional parameters, including glomalin fractions, phosphatase activity, total phenols, flavonoids and phenylalanine ammonia-lyase, and water status, supported by multivariate statistical analyses (PCA, correlation), offering a holistic understanding and practical agricultural insights.

## Conclusions

It is clear from the results that chickpea plants are severely hampered by salt stress because it interferes with important physiological and biochemical functions including growth, membrane integrity, water status and carotenoids content. AMF and proline reinforce plants’ tolerance to salinity by augmenting growth, osmoprotectants, the photosynthetic pigments and the antioxidant defense mechanisms. These findings underscore the potential of AMF and proline to advance chickpea resilience in saline environments. Furthermore, understanding the complex interplay between endogenous mycorrhizal fungi and proline metabolism in chickpea plants could inform future breeding programs and integrated crop management practices aimed at developing salt-tolerant cultivars and ensuring food security in salt-affected regions.

### Limitations and future perspectives

The present study’s primary reliance on standard physiological and biochemical markers to evaluate the function of AMF and proline applied separately in reducing salt stress in chickpeas is one of its restrictions. Firstly, the underlying molecular or mechanistic basis of the observed effects is not entirely revealed by these indicators, despite the fact that they offer insightful information on plant responses in saline environments. Therefore, in addition to comprehensive enzymatic activity assays to capture dynamic metabolic modifications, future research should combine transcriptome and proteomic techniques to uncover important stress-responsive genes and regulatory mechanisms controlled by AMF symbiosis. Secondly, the ecological significance and usefulness of AMF inoculation in chickpea agriculture under salt stress would be strengthened by combining these molecular insights with field-based experiments. Also, the lack of a combined treatment of AMF and proline prevents us from evaluating potential synergistic or additive interactions between the two. A deeper comprehension of how these biological and biochemical tactics might work in conjunction to improve stress tolerance could be obtained by such an integrated approach. Therefore, future research should concentrate on combining treatments, as this could provide more comprehensive and practical techniques to enhance chickpea performance in saline environments. Also, the mechanistic depth of AMF studies will be expanded, which will aid in the creation of long-term plans for crop resilience in soils impacted by salt.

## Data Availability

The relevant datasets supporting the results of this article are included within the article.

## Abbreviations

AMF: Arbuscular mycorrrhizal fungi
MI: Membrane injury
MLP: Membrane lipid peroxidation
MSI: Membrane stability index
ROS: Reactive oxygen species
Wc: Water content
Wsd: Water saturation deficit
